# Clinical evaluation of carbon fiber reinforced carbon endodontic post, glass fiber reinforced post with cast post and core: A one year comparative clinical study

**DOI:** 10.4103/0972-0707.48841

**Published:** 2008

**Authors:** GA Preethi, M Kala

**Affiliations:** Department of Conservative Dentistry and Endodontics, Government Dental College and Research Institute, Bangalore - 560 002, India

**Keywords:** Carbon fiber reinforced post, glass fiber reinforced post, scotch bond multi-purpose plus, RelyX ARC, post crowns, endodontics, cast post and core, carbon fiber reinforced posts, glass fiber posts

## Abstract

**Aim:**

Restoring endodontically treated teeth is one of the major treatments provided by the dental practitioner. Selection and proper use of restorative materials continues to be a source of frustration for many clinicians. There is controversy surrounding the most suitable choice of restorative material and the placement method that will result in the highest probability of successful treatment. This clinical study compares two different varieties of fiber posts and one cast post and core in terms of mobility of crown margin under finger pressure, recurrent caries detected at the crown margin, fracture of the restoration, fracture of the root and periapical and periodontal pathology requiring crown removal over the period of 12months as evaluated by clinical and radiographical examination.

**Materials and Methods:**

30 root canal treated, single rooted maxillary anterior teeth of 25 patients in the age range of 18–60 years where a post retained crown was indicated were selected for the study between January 2007 and August 2007; and prepared in a standard clinical manner. It was divided into 3 groups of 10 teeth in each group. After post space preparation, the Carbon fiber and Glass fiber reinforced posts were cemented with Scotch bond multipurpose plus bonding agent and RelyX adhesive resin cement in the first and second groups respectively. The Cast post and cores were cemented with Zinc Phosphate cement in the third group. Following post- cementation, the preparation was further refined and a rubber base impression was taken for metal-ceramic crowns which was cemented with Zinc Phosphate cement. A baseline periapical radiograph was taken once each crown was cemented. All patients were evaluated after one week (baseline), 3 months, 6 months and one year for following characteristics mobility of crown margin under finger pressure, recurrent caries detected at the crown margin, fracture of the restoration, fracture of the root and periapical and periodontal pathology.

**Results:**

Results after 12 months showed that none of the restorations among groups of cast post and core, carbon fiber reinforced post and glass fiber reinforced post with composite core restorations failed in terms of recurrent caries detected at the crown margin, fracture of the restoration, fracture of the root and periapical and periodontal pathology. One case of cast post and core and one case of carbon fiber reinforced post with composite core restorations showed slight mobility of crown margin under finger pressure at 12^th^ month recall but all the cases of glass fiber post with composite core restorations did not show any signs of mobility of crown margin under finger pressure at all the recall periods on clinical and radiographical examination.

**Conclusion:**

From this 12 months clinical evaluation of all the cases in the 3 groups comprising of cast post and core; carbon fiber reinforced post with composite core and glass fiber reinforced post with composite core restored with porcelain fused to metal crowns, it is concluded that glass fiber reinforced post with composite core when used in single rooted upper anterior teeth are associated with a higher success rate in restoration of endodontically treated teeth.

## INTRODUCTION

Root-filled anterior teeth have traditionally been restored with cast or wrought metal posts and cores. Some researchers have suggested that as these metallic materials have much higher moduli than that of the supporting dentine, this mismatch in the moduli could lead to stress concentrating in the cement lute, leading to its failure. This has led to a search for a plastic-based material that has a modulus closer to that of dentine.[1]

Recently, several new types of post material have been introduced, including carbon fiber, glass fiber etc.

Carbon fiber has certain properties that make it potentially useful in dentistry. It is biocompatible, corrosion-resistant, and strong. Most reports of the potential uses of carbon fiber in dentistry are limited to the reinforcement of existing restorative materials and as possible post material. The carbon fiber post is reported to have a modulus of elasticity that is nearly identical to that of dentine, so that it causes less tooth stress and hence, fewer root fractures. By comparison, the moduli of elasticity for stainless steel and titanium are roughly 20 and ten times greater than dentine's respectively. Posts with high moduli of elasticity do not flex with the tooth under loading, and are empirically believed to cause root fracture.[2]

The use of carbon fiber posts, preformed metallic posts, and/or custom-cast metallic casts in the anterior region has been reported to result in unsatisfactory aesthetics. As a result, aesthetic fiber posts have become more popular. They are also well accepted because of their favorable physical properties and biocompatibility. Aesthetic fiber posts can be bonded within root canal spaces with polymer dentine-bonding agents and resin cements of similar flexibility. They effectively transmit stresses between the post and the root structure, thus reducing stress concentration and preventing fracture.[3]

Glass fiber-supported resin dowel systems were introduced in 1992. The dowels are composed of unidirectional glass fibers embedded in a resin matrix that strengthens the dowels without compromising the modulus of elasticity. Another advantage of glass fibers is that they distribute stress over a broad surface area, increasing the load threshold at which the dowel begins to show evidence of microfractures. Fiber-reinforced dowels are reported to reduce the risk of tooth fractures and display higher survival rates than teeth restored with Zirconia dowels.[4]

This study reports a prospective clinical trial comparing the success rate of carbon fiber-reinforced carbon (CFRC) endodontic posts and glass fiber-reinforced posts with cast posts and cores.

## MATERIALS AND METHODS

Patients reporting to the Department of Conservative Dentistry and Endodontics for regular treatment in the Government Dental College and Research Institute, Bengaluru, were selected for the study. The study was conducted in accordance with all the local regulations for the ethical treatment of human subjects.

### Age group

The median age of the patients was 30.1 years, while the range was 18–60 years.

The selection criteria for inclusion in the study were:

- Single-rooted maxillary anterior teeth where a post-retained crown was clinically indicated.
- All teeth should have adequate root filling with no evidence of any periapical pathology, perforation, or root fracture.
- The periodontium was examined using a Williams probe using four probing sites per tooth, and patients were only included in the trial when the periodontium was stable with no evidence of bleeding on probing and a minimum of 75% bone support.

### Negative criteria

- Teeth with pulpal and periapical pathosis and those that could be potential abutments for fixed or removable prostheses were excluded.
- Patients were excluded if there was a lack of adequate posterior support, defined as the absence of all molar teeth, or if there was any obvious occlusal interference or fremitus affecting the tooth to be restored.

Twenty-five patients with the chief complaint of fractured maxillary anterior teeth were enrolled for this clinical study. Thirty root canal treated teeth in 25 patients were divided into three groups of ten teeth each:

- Group I consisted of ten cases restored with cast posts and cores.
- Group II consisted of ten cases restored with carbon fiber-reinforced posts and composite resin cores.
- Group III consisted of ten cases restored with glass fiber-reinforced posts and composite resin cores, followed by a full coverage restoration (porcelain-fused-to-metal) from January 2007 to August 2007.

### Methodology for cast post and core

Excess guttapercha was removed from the root canal using heated instruments and gates glidden drills. This was followed by space preparation by using increasing sizes of peeso reamers. A minimum apical seal of 5 mm of guttapercha filling was retained in the apical root portion. The teeth were prepared in a standard clinical manner with rotary instruments by a single operator. The root canal was then dried and lubricated with petroleum jelly. The wax pattern was fabricated for the post and core using inlay wax, and then forwarded to the laboratory for casting. The finished and polished cast post and core made of nickel-chromium alloy was then cemented into the canal using zinc phosphate cement. Following cementation of the post, the preparation was further refined and polyvinyl siloxane putty and light body impressions were taken for the laboratory construction of porcelein-fused to-metal crowns. The crown was cemented with zinc phosphate cement.

### Methodology for fiber post

The guttapercha was removed to maintain a 4–5 mm apical seal. The canal was accessed and irrigated with a solution of EDTA and sodium hypochlorite. The length of the post in the canal was determined and marked and the trimmed post was replaced in the canal to confirm its length. The prepared canal was etched with 37% phosphoric acid and after 15 seconds, the etchant was gently rinsed with water and dried with paper points. An adhesive primer and bonding agent (scotch bond multipurpose plus) was applied to the canal and an adhesive resin cement, Rely-X, was dispensed into the canal. The endodontic post was seated in the canal and the adhesive resin cement was light-cured for 60 seconds. The core was established with the successive addition of composite resin; the completed core build-up permitted prosthetic preparation. The core and the adjacent preparation were recorded in a rubber base impression material and the shade was selected, all of which were forwarded to the laboratory. Porcelain-fused-to-metal crowns were fabricated and the crowns were then cemented with zinc phosphate. An intraoral periapical radiograph was taken after each crown had been cemented. Patients were educated about oral hygiene and motivated to use proper brushing techniques.

### Postoperative evaluation

All patients were evaluated after one, three, and six months, and one year's interval. A periapical radiograph of each tooth was taken at each recall and an outline of clinical performance was recorded. Success or failure of the restorative tooth complex was evaluated clinically and radiographically, which was judged to have failed if each post crown satisfied one or more of the following criteria:

- Movement of the crown margin under finger pressure
- Recurrent caries detected at the crown margin
- Fracture of the restoration
- Fracture of the root
- Periapical or periodontal pathology requiring crown removal

The success rate of the restoration was then evaluated statistically.

## RESULTS

### Movement of the crown margin with application of finger pressure

One cast post and core restoration showed signs of movement of the crown margin with the application of finger pressure at the one year recall point; the success rate was 90% [Table 1]. One of the carbon fiber-reinforced posts and composite resin core restorations also showed slight movement of the crown margin with finger pressure at the one year recall point, thus also showing the success rate of 90% [Table 2]. However, none of the glass fiber-reinforced post and composite resin core restorations showed any signs of movement of the crown margin with finger pressure at any interval in the entire recall period, showing a 100% success rate [Table 2].

**Table 1:** Mean age distribution of study population

Material	*N*	Mean age	Std. deviation	Minimum	Maximum
Cast post and core	10	30.1	4.19	20	40
Carbon fiber-reinforced post and composite core	10	30.8	3.69	20	39
Glass fiber-reinforced post and composite core	10	31.9	4.28	20	40

**Table 2:** Clinical evaluation of mobility of crown margin under finger pressure

Months	Cast post and core		Carbon fiber-reinforced post and composite core		Glass fiber-reinforced post and composite core	
	Present	Absent	Present	Absent	Present	Absent
One	0	10 (100)	0	10 (100)	0	10 (100)
Three	0	10 (100)	0	10 (100)	0	10 (100)
Six	0	10 (100)	0	10 (100)	0	10 (100)
Twelve	1 (10)	9 (90)	1 (10)	9 (90)	0	10 (100)

Figures in parentheses are in percentages.

### Recurrent caries detected at crown margin

None of the restorations of cast posts and cores, carbon fiber-reinforced posts with composite cores, or glass fiber-reinforced posts with composite cores exhibited any recurrent caries at the various time intervals; thus, the success rate of 100% [Table 3].

**Table 3:** Clinical evaluation of recurrent caries detected at the crown margins

Months	Cast post and core		Carbon fiber-reinforced post and composite core		Glass fiber-reinforced post and composite core	
	Present	Absent	Present	Absent	Present	Absent
One	0	10 (100)	0	10 (100)	0	10(100)
Three	0	10 (100)	0	10 (100)	0	10 (100)
Six	0	10 (100)	0	10 (100)	0	10 (100)
Twelve	0	10 (100)	0	10 (100)	0	10 (100)

Figures in parentheses are in percentages.

### Fracture of the restoration

None of the restorations of cast posts and cores, carbon fiber-reinforced posts with composite cores, or glass fiber-reinforced posts with composite cores exhibited any signs of fracture of the restoration on clinical and radiographic examinations at the various time intervals, thus, the success rate was 100% [Table 4].

**Table 4:** Clinical evaluation of fracture of the restoration

Months	Cast post and core		Carbon fiber-reinforced post and composite core		Glass fiber-reinforced post and composite core	
	Present	Absent	Present	Absent	Present	Absent
One	0	10 (100)	0	10 (100)	0	10 (100)
Three	0	10 (100)	0	10 (100)	0	10 (100)
Six	0	10 (100)	0	10 (100)	0	10 (100)
Twelve	0	10 (100)	0	10 (100)	0	10 (100)

Figures in parentheses are in percentages.

### Fracture of the root

None of the restorations of cast posts and cores, carbon fiber-reinforced posts with composite cores, or glass fiber-reinforced posts with composite cores exhibited any signs of fracture of the root on clinical and radiographic examinations at the various time intervals, thus, the success rate was 100% [Table 5].

**Table 5:** Clinical evaluation of fracture of the root

Months	Cast post and core		Carbon fiber-reinforced post and composite core		Glass fiber-reinforced post and composite core	
	Present	Absent	Present	Absent	Present	Absent
One	0	10 (100)	0	10 (100)	0	10 (100)
Three	0	10 (100)	0	10 (100)	0	10 (100)
Six	0	10 (100)	0	10 (100)	0	10 (100)
Twelve	0	10 (100)	0	10 (100)	0	10 (100)

Figures in parentheses are in percentages.

**Table 6:** Clinical evaluation of periapical or periodontal pathology

Months	Cast post and core		Carbon fiber-reinforced post and composite core		Glass fiber-reinforced post and composite core	
	Present	Absent	Present	Absent	Present	Absent
One	0	10 (100)	0	10 (100)	0	10 (100)
Three	0	10 (100)	0	10 (100)	0	10 (100)
Six	0	10 (100)	0	10 (100)	0	10 (100)
Twelve	0	10 (100)	0	10 (100)	0	10 (100)

Figures in parentheses are in percentages.

### Periapical or periodontal pathology requiring crown removal

None of the restorations of cast posts and cores, carbon fiber-reinforced posts with composite cores, or glass fiber-reinforced posts with composite cores exhibited any signs of periapical or periodontal pathology upon clinical and radiographic examinations at the various time intervals, thus, the success rate was 100% [Table 6].

## DISCUSSION

The restoration of endodontically treated teeth with fiber-reinforced post systems has been drawing the attention of a growing number of clinicians. The progress in the technology of fiber-reinforced materials addressing the structure, shape, and optical properties of the posts has led to the development of materials that have overcome some of the limitations of metallic posts (platinum, alloys, or titanium) concerning aesthetic appearance, mode of failure, and clinical performance.[5]

Restorations involving endodontic posts have been investigated in quite a few clinical studies in which the causes of failure have been recorded. From these studies, it appears that the main causes of failure were identified as: caries, loss of retention of the post, loss of retention of the crown, root fracture, post distortion, and post fracture.[6]

Clinically, it is well established that the longevity of root-post-core-crown systems used to restore an endodontically treated tooth is affected by many factors, *e.g*., the design, length and thickness of the post, the ferrule effect, cementation, and the quantity of the remaining tooth substance. Many *in vitro* studies have shown that fiber-reinforced composite posts might possess some benefits over metal posts due their moduli of elasticity being closer to that of dentine. This phenomenon of 'modulus compensation of stress-induced root fractures' could have an impact on the post-core-crown restorations in the future. However, it should be noted that the modulus of the material is only one parameter influencing stress formation. Among others, the diameter of the post should also be taken into account.[7]	

Dean *et al*. carried out an *in vitro* comparison of carbon fiber with conventional cast posts and found that no root fractures were associated with carbon fiber posts, whereas with cast posts, 50% of the teeth had root fractures.[1]

In studies on the retention of carbon fiber-reinforced composite posts, luting procedures have been reported as the critical point, with failures occurring at the post-cement and post-core junctions. Moisture and water contact are problems to all resin-based systems, inducing hydrolysis and degradation of the organic matrix. As the matrix swells due to water or thermal changes, hydrothermal stresses are induced at the fiber/matrix interface that can result in debonding and/or matrix cracking, which facilitates further water absorption.[8]

A retrospective study of 236 patients evaluated the treatment outcome of teeth restored with the composipost system (carbon fiber-reinforced epoxy resin posts). Out of the 236 teeth restored with carbon fiber-reinforced epoxy resin posts, neither clinical nor radiographic examination revealed any dislodgement or root or post fractures. Promising results after 2–3 years of clinical service indicate that this system can be a viable alternative to conventional post-and-core systems.[9]

Three factors influence the durability of restorations against recurrent caries: patients' caries activity, the quality of treatment provided, and the cariostatic effects of the restorative material. In the present clinical or *in vivo* study, the patients were given oral hygiene instructions, which could have affected their motivation levels. This, in turn, would have had an effect on the absence of recurrent caries in all the three groups.

According to the *in-vitro* study by Arturo Martinez-Insua *et al.*, who compared the fracture resistance of two types of restorations: teeth restored with prefabricated carbon-fiber posts and composite cores to cast dowel-core restored teeth, concluded that significantly higher fracture thresholds were recorded for the cast post and core group. Teeth restored with carbon-fiber posts and composite cores typically showed failure of the post/core interface before the fracture of the tooth occurred. This failure occurred in response to acceptably high loads. By contrast, teeth restored with cast posts and cores typically showed fracture of the tooth, albeit in response to loads that rarely occur *in vivo*.[10]

Rosentritt *et al*. compared the *in vitro* fracture strength of metallic and tooth-coloured posts and cores. Typical failure of the metal systems was marked by loosening of the bonding and pulling out of the posts in contrast to fracture of the ceramic posts. The study showed sufficient fracture strengths in all-ceramic systems and better fracture strength in posts with composite cores in comparison to gold post and core systems.

Coronal microleakage is a major cause of endodontic failure. Saliva and organisms from the mouth migrate rapidly alongside poorly adapted restorations and even root fillings that appear well condensed. The periradicular tissues will be inflamed by such reinfection and the reactivation of micro-organisms lying dormant after initial treatment. A well sealing coronal restoration is therefore critical to endodontic success.

In this one year, clinical evaluation of all the cases in the three groups comprising of cast posts and cores, carbon fiber-reinforced posts with composite cores, and glass fiber-reinforced posts with composite cores restored with porcelain-fused-to-metal crowns at one, three, and six months, and one year recall visits showed that glass fiber-reinforced posts with composite core restorations resulted in the highest success rate. The cast post and core restorations and carbon fiber-reinforced posts with composite core restorations showed 10% failure rates with relation to movement of the crown margin under finger pressure. With respect to recurrent caries detected at the crown margin, fracture of the restoration, fracture of the root, and periapical or periodontal pathology requiring crown removal, all the cases of cast posts and cores, carbon fiber-reinforced posts with composite cores, and glass fiber-reinforced posts with composite cores restored with porcelain-fused-to-metal crowns showed 100% success rate at all the recall visits up to 12 months.

## CONCLUSION

From this clinical evaluation over a period of one year of all the cases in the three groups comprising of cast posts and cores, carbon fiber-reinforced posts with composite cores, and glass fiber-reinforced posts with composite cores restored with porcelain-fused-to-metal crowns, it can be concluded that when used in single-rooted, upper anterior teeth, glass fiber-reinforced posts with composite core are associated with a higher success rate in the restoration of endodontically treated teeth.
